# Hypoxia Pathway Proteins in Normal and Malignant Hematopoiesis

**DOI:** 10.3390/cells8020155

**Published:** 2019-02-13

**Authors:** Ben Wielockx, Tatyana Grinenko, Peter Mirtschink, Triantafyllos Chavakis

**Affiliations:** Institute of Clinical Chemistry and Laboratory Medicine, Technische Universität Dresden, 01307 Dresden, Germany; tatyana.Grinenko@ukdd.de (T.G.); Peter.Mirtschink@ukdd.de (P.M.); Triantafyllos.chavakis@ukdd.de (T.C.)

**Keywords:** oxygen sensors, hypoxia, hematopoietic stem cells, leukemia, HIF

## Abstract

The regulation of oxygen (O_2_) levels is crucial in embryogenesis and adult life, as O_2_ controls a multitude of key cellular functions. Low oxygen levels (hypoxia) are relevant for tissue physiology as they are integral to adequate metabolism regulation and cell fate. Hence, the hypoxia response is of utmost importance for cell, organ and organism function and is dependent on the hypoxia-inducible factor (HIF) pathway. HIF pathway activity is strictly regulated by the family of oxygen-sensitive HIF prolyl hydroxylase domain (PHD) proteins. Physiologic hypoxia is a hallmark of the hematopoietic stem cell (HSC) niche in the bone marrow. This niche facilitates HSC quiescence and survival. The present review focuses on current knowledge and the many open questions regarding the impact of PHDs/HIFs and other proteins of the hypoxia pathway on the HSC niche and on normal and malignant hematopoiesis.

## 1. Introduction: Hematopoietic Stem Cells in the Hypoxic Bone Marrow Environment

Hematopoiesis (i.e., the generation of blood cells) relies on hematopoietic stem cells (HSCs), a rare cell population in the bone marrow [1]. HSCs are quiescent cells at the top of a tree-like hierarchy and support homeostatic hematopoiesis, thereby meeting the demands of physiological blood cell turnover as well as regeneration in the context of hematopoietic stress [2,3,4]. The capacity of HSCs to generate new HSCs upon division, a process designated as self-renewal, together with their ability to generate all blood cell types, defines their stemness [5]. The unique metabolic properties of HSCs allow them to maintain this stemness in an environment of low cell cycling and metabolic dormancy, which, in turn, is required to preserve their genomic integrity and function [6]. In adults, quiescent HSCs reside within a specialized microenvironment of the bone marrow (BM), whereby HSCs are located in close vicinity to endothelial cells (ECs) [7,8], perivascular cells [7], mesenchymal stem cells [9] and megakaryocytes [10,11], which are all considered as the BM niche.

The entire BM, including the niche microenvironment, displays low oxygen pressure; the O_2_ levels in the BM of live animals lie between ~7 and 31 mmHg or 1 and 4% [12]. In comparison, pO_2_ levels in the peripheral blood in humans lie in the range of 75–100 mmHg (10–13%). Hence, HSCs experience chronic hypoxia; consistently, their quiescence relies on anaerobic glycolysis and a relatively low production of reactive oxygen species (ROS) [13]. It was considered for years that hypoxic HSCs reside in the poorly perfused endosteal zone at a certain distance from oxygen-rich blood vessels [14]. Interestingly, the highest partial oxygen pressure is found in arteriole-rich endosteal regions. According to the current perception, HSCs and progenitor cells (HSPCs) reside in heterogeneous peri-sinusoidal and peri-arteriolar niches with comparable low oxygen profiles irrespective of their exact location within the BM [12,15]. 

The present review emphasizes recent advances in the role of the hypoxia response in HSPC metabolism and function as well as in the crosstalk between HSPCs and the BM niche. We here review these aspects both in the context of normal hematopoiesis/normal HSCs and hematological malignancies/leukemic stem cells (LSCs).

## 2. Hypoxia Pathway Proteins

The organism’s adaptation to variable oxygen levels is tightly regulated by the cellular hypoxia pathway. The enhanced activation of the transcription factor family of hypoxia-inducible factors (HIFs) is the primary response to reduced oxygen levels [16]. HIFs are heterodimers comprising two basic helix-loop-helix proteins (the HIFα and HIFβ subunits), which recognize the hypoxia response element (HRE, entailing the G/ACGTG motif) in the promoter of several genes, with major roles in different cell functions, including cellular metabolism, survival and proliferation, and in processes, such as angiogenesis, hematopoiesis and inflammation [17]. Whereas the expression of HIF1α protein is ubiquitous, the cellular expression of the HIF2α subunit is rather restricted to certain cells, for instance, cells of hematopoietic origin, ECs, adipocytes, glial cells and interstitial cells of the kidney [18,19,20,21,22,23]. While there is some functional overlap of both HIF isoforms, HIF1α and HIF2α also have distinct functions, each specifically regulating different genes. It was recently suggested that, despite the identical consensus DNA recognition sequence, each HIF heterodimer (HIF1α or HIF2α + HIFβ) interacts with cell-type-specific HREs throughout the entire genome with its own binding distributions, thus supporting a potential conserved mechanism that distinguishes HIF1 from HIF2 sites at the DNA [24]. 

HIFβ, which binds to both HIFα subunits, is constitutively expressed, whereas the HIFα subunits are regulated at the protein level in an oxygen-dependent manner. In normoxic conditions (physiological oxygen concentrations), HIFα expression is suppressed by a mechanism that involves hydroxylation of either an asparagine or two specific proline residues [25], by the factor inhibiting HIF (FIH) enzyme or HIF prolyl hydroxylase domain (PHDs) enzymes, respectively. The former hydroxylation diminishes the transcriptional activity of HIF, while the latter modification promotes proteasomal degradation of HIF via the von Hippel–Lindau tumor suppressor protein (pVHL) [26,27,28,29]. As the co-substrates O_2_, Fe(II), ascorbate and 2-oxoglutarate (2-OG) are required for PHD function and hence for HIF hydroxylation, PHDs are less operational under hypoxic conditions. Therefore, HIFα is protected from degradation under hypoxia and HIFα subsequently translocates to the nucleus, where it exerts its activity, directly promoting the transcription of more than 1000 genes [30,31]. 

**Figure 1:**
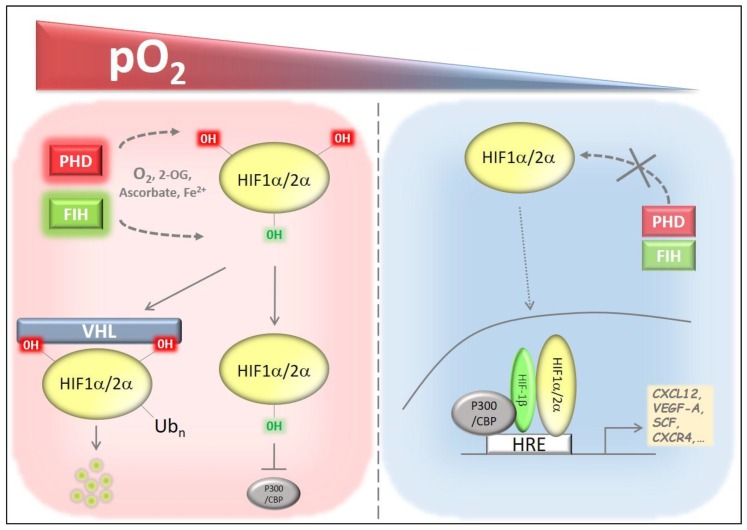
Oxygen-dependent regulation of HIFα and its target genes. HIFα is continuously hydroxylated by PHDs and FIH in sufficiently oxygenated environments (left). Hydroxylation of two proline residues by PHDs leads to subsequent proteasomal degradation after binding with VHL, whereas asparagine hydroxylation by FIH inhibits the interaction of HIF with p300/CBP and prevents transcriptional activation. Under hypoxia (right), HIFα is stabilized and translocates to the nucleus, binding to HIFβ as well as other co-factors, which promotes the transcriptional activation of target genes that harbor HRE sequences in their promoter region (HIF: hypoxia-inducible factor, FIH: factor inhibiting HIF, PHD: prolyl hydroxylase domain, VHL: von Hippel–Lindau, CBP: CREB-binding protein, HRE: hypoxia responsive element).

The PHD family consists of three members, all of which are able to hydroxylate both HIFα subunits (Figure 1). Despite their high sequence homology at their C-terminal domains, their N-terminal domains as well as their subcellular localization and tissue expression pattern are distinct [32], suggesting different functions for all three PHDs. In fact, each PHD isoform hydroxylates a particular HIFα subunit with a different affinity. In normoxia, PHD1 and PHD2 preferentially degrade HIF2α and HIF1α, respectively. In turn, HIF2α is the preferred substrate of PHD3 under hypoxic conditions [33]. Interestingly, *PHD3* is a target gene of HIF1α; hence, the induction of PHD3 in hypoxia provides a loop for the downregulation of HIF2α [34]. Despite the major role of PHDs in the HIF-pathway, these oxygen sensors have also been associated with the regulation of other central proteins, including TGFβ and IκB kinase (IKKβ), a major regulator of the nuclear factor-kappa B (NF-κB) pathway ([35,36,37,38,39]). Consequently, PHDs have a differential impact on development and adult life homeostasis. Whereas the complete deletion of PHD1 or PHD3 does not lead to lethality, PHD2 deficient embryos die between E12.5 and 14.5 due to cardiac malfunction and defects in the placenta vasculature [20,40,41]. Frankly, PHD2 is considered to be the key oxygen sensor, and its function has been associated with several different physiological and pathological settings (comprehensively reviewed by our group in [17]). In adults, full PHD1 deficiency leads to a clear shift in cellular metabolism from oxidative to glycolytic bioenergetics in the skeletal muscle [42], and to increased hepatocyte proliferation [43]. PHD3 deficient mice exhibit altered innervation and reduced blood pressure at rest in the central nervous system [44].

## 3. Hypoxia Pathway Proteins in Normal Hematopoiesis

In the last two decades, numerous studies have indicated the importance of HIFs in HSPC maintenance. The hypoxic environment of the HSC niche obviously predisposes for a contribution of HIFs to hematopoiesis and, in particular, to the preservation of the functionality of the HSC. In vitro experiments with hypoxic culture conditions demonstrated that the hypoxia-facilitated maintenance of HSC quiescence and to some extent even the self-renewal of HSPCs was preserved. Hence, hypoxia is a functional component of the sites in which stem cells are preserved [45]. This notion was further supported by histological studies demonstrating the selective labeling of HSPCs in the BM with “pimonidazole”, a probe indicating intracellular hypoxia [15,46], as well as from the stable expression of HIF1α and the functional role of HIF in primitive hematopoietic populations [25,47,48]. In fact, a functional link between stemness, reduced oxygen availability and HIFs has been suggested for multiple stem cell types and extensively studied in the case of HSPCs [49,50]. The inactivation of HIF1α in mouse HSCs, through the use of a mouse line with inducible Mx1:Cre and conditional *HIF1α* allele, resulted in loss of HSC quiescence. Conversely, the stabilization of HIF1 in HSCs, via the inhibition of VHL, stimulates anaerobic glycolysis in a manner that depends on the pyruvate dehydrogenase kinase 1 (PDK1). Furthermore, the latter enzyme inhibits the mitochondrial function in HSCs by limiting the influx of glycolytic metabolites, which appeared to be essential for HSC homeostasis and self-renewal potential [25,48]. Using a conditional mouse line with the inactivation of PHD2 in different cells, including cells of the hematopoietic system, we found HIF1α-dependent increased self-renewal of multipotent progenitors, but not of CD34^−^ HSCs. The repopulation potential of PHD2-deficient HSPCs was greatly hampered, as assessed by competitive transplantation studies; in contrast, no difference was detected in the repopulation potential of HSCs and the earliest multipotent progenitors [51]. However, as opposed to the results obtained by Takubo and colleagues [25], our PHD2-deficient hematopoietic precursors displayed no diminished homing to the BM, suggesting an alternative mechanism for the observed decreased peripheral and central chimerism. Subsequent findings by Vukovic et al. revealed no effect of HIF1α deficiency in HSCs on their survival or their ability to reconstitute long-term, multi-lineage hematopoiesis. Moreover, loss of HIF1α did not affect HSC self-renewal after serial transplantation assays or as a response to hematopoietic injury [52]. Potential explanations for the discrepancy between the aforementioned studies by Takubo et al., Vukovic et al. and our study [25,51,52] may be the significant differences in the models used for the induction of gene ablation in the hematopoietic system and the targeting of different components of the hypoxia response pathway (e.g., Mx1:Cre-HIF1α^f/f^, Mx1:Cre-VHL^f/f^ or CD68:Cre-PHD2^f/f^), as well as the timing of the deletion. Only Vukovic et al. avoided non-hematopoietic effects of the Cre-recombinase system used, as they induced HIF1α deletion (by the activation of the Mx1:Cre recombinase) following HSC transplantation, thereby selectively deleting HIF1α only in hematopoietic cells. Therefore, differential deletion of the targeted factors of the HIF pathway in other cellular components of the BM microenvironment might also affect the functionality of HSPCs, thereby providing a possible explanation for the aforementioned discrepancies.

HIF2α activity in HSPCs is dispensable, as the deletion of this transcription factor, even in combination with HIF1α deficiency, did not result in alterations of the HSC fractions under steady-state and/or hematopoietic stress in mice [53]. In contrast, Rouault-Pierre et al. demonstrated decreased long-term repopulating activity of human cord blood CD34^+^ stem cells upon HIF2α silencing, associated with higher levels of ROS in HIF2α-deficient HSPCs, which resulted in endoplasmatic reticulum (ER) stress and cell death [54]. A recent study showed that hematopoietic cell-specific deletion, mediated via Vav:Cre, of the HIFα dimerization partner HIFβ (also named ARNT) induced apoptosis in long-term-repopulating HSCs (LT-HSCs) but no alterations in their cell cycle—a phenotype most likely linked to the loss of BCL2 and VEGFA expression. Strikingly, the hematopoietic cell-specific deletion of both HIF1α and HIF2α phenocopied key aspects of the HSC alterations due to ARNT deficiency [55]. 

Taken together, the complexity of the results from the aforementioned studies suggest that further investigations are warranted, which should focus not only on the role of hypoxia pathway proteins in HSPCs, but also in cell populations of the BM microenvironment, located in proximity to the HSPCs. Indeed, the activity of HIFs/PHDs in cellular components of the HSC niche, including (peri-)vascular cells, osteoblasts, mesenchymal stem cells (MSCs) and even megakaryocytes and macrophages, might be critical for the positioning and function of HSCs. 

## 4. The BM Niche and Hypoxia

**Figure 2:**
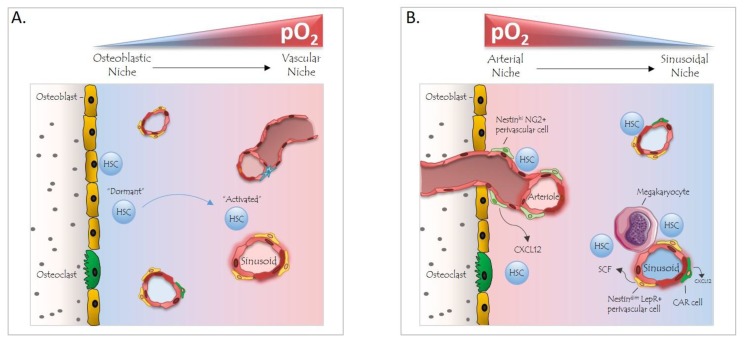
Models of hypoxic hematopoietic stem cell (HSC) niches in the bone marrow. (**A**) Initially, the hypoxia niche model suggested that dormant HSCs were found in a poorly perfused osteoblastic/endosteal niche. Deepest hypoxia was thought to be present in areas distant from vascular structures and HSCs were oxygenated and activated upon approaching oxygen-sufficient blood vessels. (**B**) Recently, new studies have led to the finding that the bone marrow (BM) cavity exhibits a different oxygen gradient than originally thought, with the highest pO_2_ in arteriole-rich endosteal zones and the lowest in sinusoidal areas of the BM. HSCs and progenitor cells (HSPCs) may reside in heterogeneous perivascular niches (perisinusoidal and periarterial).

The function of cells of the BM niche with respect to the regulation of quiescence, proliferation and differentiation of HSPCs has been the topic of extensive investigations and scientific discussions in the past decade. Recent studies from several groups support the idea that two distinct types of functional vascular niches exist: (i) the arteriolar niche, which is close to the endosteum and contains quiescent HSCs that are in close vicinity to so-called Nestin^hi^/NG2^+^ pericytes (cells surrounding the vascular endothelium in an intertwined fashion and exerting a vessel-supportive function); and (ii) the sinusoidal niche, containing proliferative HSCs adjacent to Nestin^dim^/Leptin Receptor^+^ (LepR) stromal cells [7,56,57,58,59,60]. However, it has also been postulated that arteriole-associated NG2^+^ pericytes do not exert a niche function and that HSC distribution in the BM may be random [56]. Arguments against the hypothesis on random HSC distribution were provided by the association of an HSC subset with and its functional regulation by megakaryocytes [11,61], as well as by the identification of quiescent HSCs with low ROS levels in the proximity of arterioles and megakaryocytes [62]. The aforementioned studies have led to a revision of the original model of a hypoxic niche that suggested HSCs to reside in a poorly perfused osteoblastic niche with deepest hypoxia, distant from any vascular structures [63]. On the other hand, the revised model relies on the existence of an oxygen gradient in the BM cavity, with the highest partial O_2_ pressure in the arteriolar niche of the endosteal zone and the lowest O_2_ concentration in deeper areas of the BM, where the sinusoidal niche is located (Figure 2). Nevertheless, more studies are required to better define niche structures and respective locations and functions of the heterogeneous population of HSCs. 

Important recent insights were gained on the differential impact of two niche factors expressed by perivascular cells, the chemokine C-X-C motif ligand 12 (CXCL12) and stem cell factor (SCF). Deficiency of CXCL12 in NG2^+^ (arteriolar) but not LepR^+^ (sinusoidal) perivascular cells resulted in dramatic changes in the number and localization of HSCs, whereas the loss of SCF only in LepR^+^ cells led to diminished HSC numbers in the mouse BM [64]. Interestingly, both CXCL12 and SCF were previously identified as hypoxia/HIF-induced proteins. In different sets of tumor cell lines, SCF was found to be induced by either HIF1α or HIF2α [65,66]. In a study by Wang et al., chromatin immunoprecipitation and promotor mutation studies convincingly demonstrated the direct transcriptional activation of SCF by an HIF2α-specific HRE in its promoter [65]. In a PHD2-deficient mouse model of pulmonary arterial hypertension and in PHD2-silenced human lung microvascular ECs, endothelial CXCL12 expression was enhanced in an HIF2α- but not an HIF1α-dependent manner [23]. Therefore, it would be of great interest to focus future HSC-related research on the modulation of the PHD/HIF system in (peri)vascular cells of the BM niche in adult mice, as it is likely that this type of transcriptional regulation of hypoxia pathway proteins may influence the quiescence and proliferation of HSCs.

## 5. Hypoxia Pathway Proteins in HSC Mobilization

The different direct and indirect biologic effects of the PHD/HIF system on HSPCs also extend to the regulation of their mobilization. Interestingly, the expansion of myeloid progenitors due to Granulocyte Colony-Stimulating Factor (G-CSF), commonly used in the clinic to elicit mobilization of HSPCs for therapeutic transplantation, is associated with enhanced abundance of hypoxia in the BM microenvironment. The latter leads to the stabilization of HIFα and the activation of the target gene *VEGFA*, which promotes HSC mobilization and increased vascular permeability [67]. Furthermore, HIF-activation is essential for HSC mobilization in response to a combination of G-CSF and the CXCR4 antagonist Plerixafor; CXCR4 is the receptor of CXCL12 and directly inducible via hypoxia/HIF. This was not only demonstrated in mice with HSC-specific HIF1α deletion but also by the administration of a broad PHD-inhibitor, which pharmacologically stabilizes HIFα [68]. 

## 6. Hypoxia Pathway Proteins in Malignant Hematopoiesis

The expression of hypoxia pathway proteins in leukemic stem cells (LSCs) and their possible biologic impact on leukemogenesis have also been explored. In this context, Wang et al. demonstrated the high expression of HIF1α at the protein level in CD34^+^CD38^−^ leukemic cells, but not in CD34^+^CD38^+^ cells. The silencing of HIF1α or inhibition with the HIF blocking agent echinomycin reduced the colony forming activity of mouse lymphoma and human myeloid acute leukemia (AML) stem cells. The mechanism involved the HIF1-dependent repression of a negative feedback loop in the Notch pathway potentially regulating the maintenance and/or self-renewal of the LSC [69]. Via HIF1α immunohistochemistry, it was shown that about 20% of normal karyotype AMLs displayed a high percentage of cytoplasmic HIF1α-expressing leukemic cells (more than 5%), which was associated with reduced event-free survival [70]. HIF1α also indirectly downregulated miR-17/20a by targeting p21 and STAT3, ultimately interfering with AML cell differentiation [71]. HIF1α may also represent an essential target within the tumor microenvironment. Although several strategies are being explored in solid tumors at the moment, including the use of novel antisense oligonucleotides against HIF1α and small-molecule HIF1α inhibitors, the potential therapeutic application of HIF1α inhibition for leukemia remains to be determined. 

To date, only a few studies have focused on HIF2α expression in leukemia. Rouault-Pierre et al. demonstrated that almost one third of a collection of primary AMLs displayed higher HIF2α levels than normal HSCs. The silencing of HIF2α expression in a selection of these leukemic blasts resulted in the significantly reduced engraftment of these cells to the BM of immune-deficient mice [54]. Additionally, human HIF2α cDNA in mouse syngeneic models of myeloid pre-leukemia resulted in enhanced lethality, whereas the silencing of the transcription factor significantly prolonged the survival of the mice. However, although the same authors found an elevated expression of HIF2α in subsets of human AML, these patients showed a tendency towards higher disease-free survival—clearly demonstrating the complexity of leukemogenesis [72].

Although the aforementioned studies suggest an important influence of HIFs on the maintenance and propagation of LSCs, different results have also been reported. In an HIF2α conditional deletion mouse line, Vukovic et al. explored the effect of HIF2α deficiency on leukemia development. Loss of HIF2α resulted in the faster development of LSCs and shortened the latency of leukemia resulting from the MLL-AF9 translocation. Interestingly, the accelerated initiation of leukemia due to HIF2α loss was further heightened by the concomitant genetic deletion of HIF1α. However, the genetic or pharmacological inhibition of HIFs in established LSCs did not affect their function or latency. Collectively, the authors concluded that HIFs may act as suppressors of leukemogenesis without necessarily regulating the maintenance of LSCs [73]. In three different murine AML models, the absence of HIF1α did not inhibit (and in certain models in fact promoted) leukemia development, hence challenging the assumption that HIF1α is a therapeutic target in AML [74]. This conclusion was also strengthened by the finding that loss of HIF1α in a murine myeloproliferative neoplasia model induced by Internal Tandem Duplications (ITD) of the *FLT3* gene (found in approximately 25% of AML cases) exacerbated disease development [75].

## 7. Concluding Remarks

Since the discovery of HIFs more than 25 years ago, numerous studies have described the influence of hypoxia and hypoxia response pathway proteins in various physiological and pathological processes. About a decade ago, the association between low oxygen tension microenvironments and the function of stem cells, including hematopoietic [25], mesenchymal [60] and neuronal stem cells, was identified [76]. Initially, HIF1α and its oxygen sensor PHD2 were considered to regulate the self-renewal capacity, quiescence and differentiation of HSPCs as well as the switch of their energy metabolism from oxidative phosphorylation to anaerobic glycolysis [48]. The functions described for HSCs were also extended to cancer stem cells of hematological malignancies [69]. Recently, however, the potential cell-intrinsic effect of HIF1α and HIF2α in HSPCs and LSCs has been challenged; this led to the hypothesis that indirect not-HSC-cell-intrinsic effects of hypoxia pathway proteins or the function of this pathway in niche cells may play a more prominent role than originally anticipated. Although it is established that the proper function of HSCs and of their downstream progenitors depends on their interaction with various BM niche cells, experiments targeting hypoxia pathway proteins in the latter compartment are still missing. In this respect, it is necessary to clarify if the expression of HIF1α and/or HIF2α, and by extension of other potential hypoxia pathway proteins in different niche cell components, contributes to the maintenance of the quiescence/stemness of HSCs and LSCs. In addition, whether hypoxia pathway proteins interact with components of the niche [77,78,79] remains to be investigated. Moreover, as the therapeutic use of small molecule inhibitors against HIFs/PHDs is currently under investigation for different disorders, it would be of importance and interest to evaluate the benefit of such therapeutic strategies with regard to BM regeneration, HSPC mobilization as well as hematological malignancies.
